# Transactions of the Mississippi State Medical Association

**Published:** 1877-10-20

**Authors:** 


### BOOK NOTICES.

TRANSACTIONS OF THE MISSISSIPPI STATE MEDICAL ASSOCIATION.—Through the courtesy of Dr. Robt. Kelly, of Jackson, we have been furnished with a copy of the *Transactions*, of the Mississippi State Medical Association, which assembled at Grenada in April last. It is a highly creditable compilation of 175 pages, neatly and tastefully gotten up. The papers reported are highly interesting and instructive.

